# Midwives’ experiences of reducing maternal morbidity and mortality from postpartum haemorrhage (PPH) in Eastern Nigeria

**DOI:** 10.1186/s12884-022-04804-x

**Published:** 2022-06-08

**Authors:** Felicity Agwu Kalu, Joan N. Chukwurah

**Affiliations:** 1 School of Nursing and Midwifery, Queen’s University Belfast, Belfast, Northern Ireland; 2 Department of Nursing Science, University of Benin, Benin City, Nigeria

**Keywords:** Postpartum haemorrhage, Active management of the third stage of labour, Antenatal anaemia, Maternal morbidity and mortality, Midwives, Respectful care, Multidisciplinary collaboration, Cultural competency, Access to maternity care

## Abstract

**Background:**

Postpartum haemorrhage (PPH) is one of the major complications of childbirth which may result in maternal morbidity and mortality, especially in low and middle-income countries like Nigeria. Midwives play a vital role in preventing and managing PPH in Nigerian rural communities. The aim of this study is to understand the experiences of midwives in rural maternity care settings in order to provide appropriate support and improve practice.

**Methods:**

An exploratory, qualitative study of a purposive sample of 15 practicing midwives was carried out using semi-structured interviews from November 2018 to February 2019. Data were transcribed verbatim and analyzed using content analysis.

**Results:**

Four themes were identified: 1. interventions for preventing PPH; 2. approaches to managing PPH; 3. challenges of preventing and managing PPH and 4. ways of supporting midwives to overcome these challenges in rural health care settings. Midwives employed various strategies, such as antenatal education, diagnosis and treatment of anaemia to counteract complications from possible PPH. Understanding PPH as a life-threatening condition enabled the midwives to provide holistic and effective management that sometimes involved a multidisciplinary team approach. Inadequate resources and delay in seeking health care, however, militate against their efforts. The midwives also identified the need for continuing education and training to enhance their standards of care.

**Conclusion:**

These midwives in Nigerian rural health care settings engage in preventive practices and active management of PPH though not without barriers, such as inadequate resources. There is a need for midwives in rural areas to have cultural competence, be provided with adequate resources and participate in ongoing education in order to be more effective.

**Supplementary Information:**

The online version contains supplementary material available at 10.1186/s12884-022-04804-x.

## Background

Pregnancy and childbirth are times of joy and celebrations for many families. These periods, however, are also challenging moments because of frightening and traumatic experiences of maternal morbidity or mortality associated with postpartum haemorrhage (PPH) or other complications [1–4]. Globally, 830 women die daily because of preventable complications of pregnancy and childbirth [1]. According to the World Health Organisation (WHO), 99% of maternal deaths occurred in low and middle-income countries (LMIC) [1, 2, 4].

The Nigerian Demographic Health Survey in 2019 showed a reduction of the maternal mortality ratio (MMR) from 814 to 556 per 100,000 live births [5]. Such a high MMR in Nigeria is quite disturbing and far from reaching the Sustainable Development Goal 3 to reduce MMR below 140 per 100, 000 live births [1, 6].

Evidence from the literature has shown that PPH is a major cause of death in LMIC such as Nigeria [3, 7–12]. Minor PPH is described as vaginal blood loss of 500–1000 ml, while major PPH is any blood loss above 1000 ml [13]. Hypovolaemic shock, multiorgan failure and maternal death can occur depending on the severity of PPH, pre-existing anaemia, untreated or ineffective management [2, 14, 15]. Prevention of anaemia in pregnancy, appropriate management of first and second stages of labour, effective physiologic or active management of the third stage of labour with uterotonic agents may prevent complications of PPH [2, 9, 12, 16–21]. Prompt diagnosis and treatment are essential and will minimize the incidence of morbidity and mortality [2, 10, 12, 19]. Delay of women to seek maternity care, delay to arrive in health care facilities and delay in receiving care from skilled health care professionals play a role in high MMR and serious morbidity in Nigeria [22–24]. Experiences of midwives involved in preventing and managing PPH in rural settings is lacking, Midwives are the most frequently available skilled maternity care providers in rural communities. Understanding their experiences in maternity care is essential in order to provide adequate support for them to enhance their skills in providing appropriate maternity care, hence the need for this study.

## Methods

### Study design

This qualitative study was carried out to explore midwives’ experiences in reducing maternal mortality and morbidity from PPH in rural Eastern Nigeria.

### Participants and recruitment

A purposive sample of 15 qualified and practicing midwives with experiences of preventing and managing PPH willingly participated in the study. An administrative staff member in the labour ward acted as gatekeeper to recruit the participants. The process of recruitment involved issuing an initial notice about the study and invitation to participate. An information leaflet was issued subsequently.

### Data collection

**Fig. 1:**
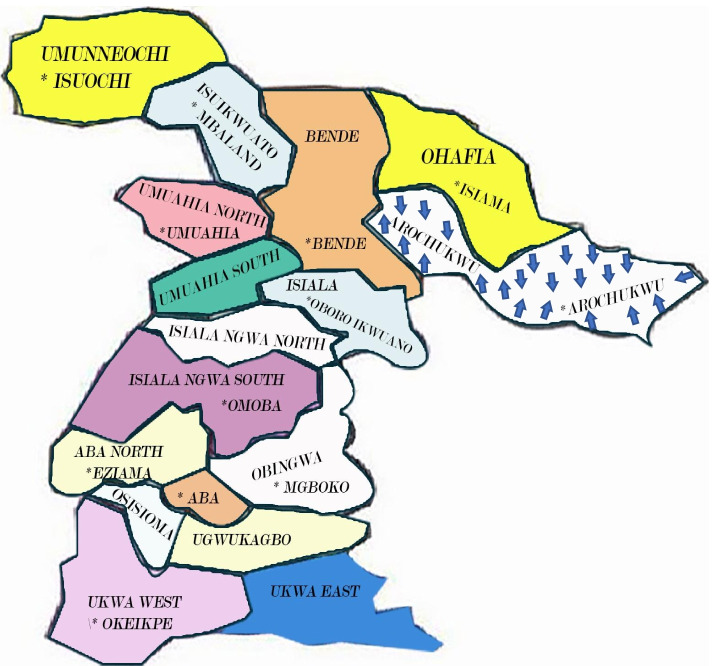
Map of Local Government Areas in Abia State including Arochukwu LGA [25]

**Fig. 2:**
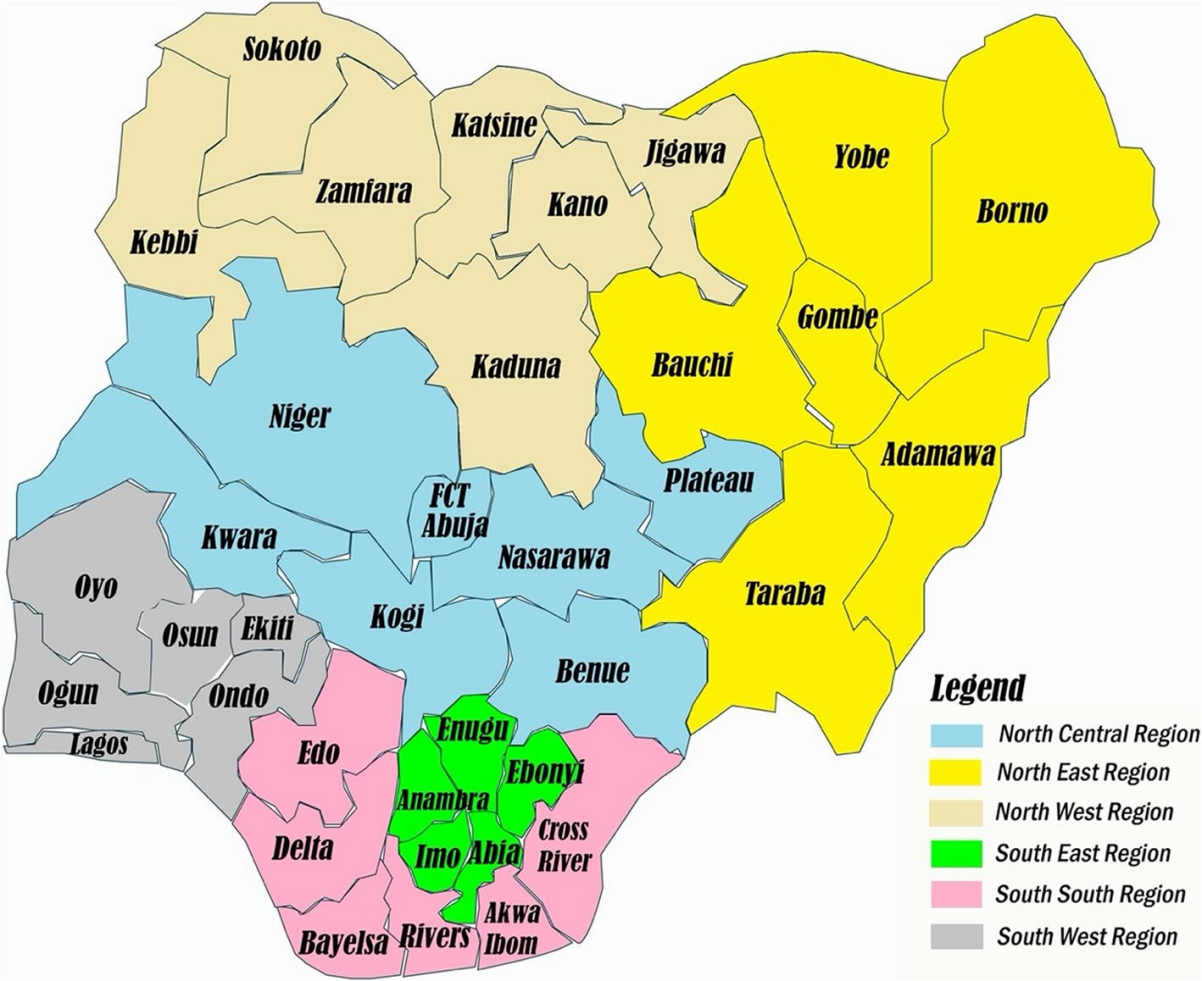
Map of Nigeria showing the six Regions, 36 States and Federal Capital Territory [26]

Data were collected by the first author (FAK) through semi-structured interviews with 15 participants from health care facilities in Arochukwu Local Government Area (LGA) of Abia State in South East Nigeria from November 2018 to February 2019. Arochukwu LGA is populated by the Ibo ethnic group. It shares boundaries with other ethnic groups such as the Ibibios in Akwa Ibom State and the Efik in Cross River State (Figs. 1 and 2) [25, 26].

The interviews took place in convenient locations chosen by the participants. An interview guide (supplementary file) was used with introductory open-ended questions such as: Please tell me about your experiences of preventing and managing PPH in your rural community. What is it like to prevent and manage PPH in your health facility? The rationale for using semi-structured interviews was to enable the participants to freely respond and discuss their experiences in their own words. These interviews also provided additional benefits of enabling the researchers to probe and clarify some points with the participants in a more flexible way. The interviews lasted between 40 and 55 min. Interviews were audio-recorded. Data were transcribed verbatim. Data saturation occurred after 15 interviews. This means that no new theme emerged. A decision was made at that point to stop collecting data. Data saturation was confirmed by both authors (FAK and JNC), who are researchers and lecturers in midwifery.

### Data analysis

Data were analysed independently by both authors using a qualitative content analysis approach as identified by Graneheim and Lundman, to provide new insights and more understanding of the experiences of midwives and to inform practical action [27–29]. The content analysis was guided by the research questions, and answers were provided through examining all texts of the interviews. The research question was: What is it like to prevent and manage PPH in a rural community in Nigeria? The transcripts were read and re-read by each author and those words and sentences containing evidence and information regarding the research question were classified as meaning units. This was followed by condensing and coding of the meaning units, which were further sorted into their corresponding categories. Finally, the underlying meaning of the categories was organized into different themes. The findings of both authors were discussed together to reach consensus. Four themes that reflected the aim of the study emerged from data analysis.

### Data trustworthiness

Data trustworthiness was achieved using Lincoln and Guba’s framework for ensuring rigor as a guide. This framework includes credibility, transferability, dependability and confirmability [30]. To achieve credibility of the study, member checking was carried out to confirm the accuracy of the themes. This means that the researchers returned to the participants individually after the interviews to confirm the accuracy of the findings. All participants confirmed that the transcripts contained accurate information. During the interviews, participants were encouraged to clearly explain the meaning of their experiences. Credibility of the study was further enhanced through reflexivity. This was achieved by the researchers’ efforts to control bias through self-awareness and by describing and interpreting their own experiences of the study as researchers [31, 32]. In relation to transferability of the study findings, the researchers have placed the study in context by providing readers with examples of other studies with similar phenomena. Dependability is about the stability of the findings over time [30]. The phenomena of interest, design and methods remained the same throughout the study to enhance dependability. Although new interviews could lead to new phenomena, use of the interview guide enabled participants to remain focused on the topic. Confirmability was achieved by ensuring credibility, transferability and dependability of the study.

### Reflexivity

The researchers kept reflective research diaries and remained engaged in critical self-awareness throughout the process to prevent bias. In addition, the research skills, their knowledge as educators in midwifery and previous roles as midwifery practitioners enabled them to be theoretically sensitive to the emerging themes. Furthermore, their professional background as midwives helped to communicate effectively with participants who were also midwives, having similar background knowledge and vocabulary.

## Results

A total of 15 midwives from Arochukwu LGA participated in the study. All participants were registered midwives and nurses. Out of the 15 midwives, six practiced in three public hospitals/secondary health facilities (out of nine hospitals), and nine in primary health facilities (out of 40 primary health facilities in the LGA). Each health facility had its own dispensary. Table 1 provides demographic characteristics of the participants. Data analysis revealed four themes: (a) Interventions for preventing PPH; (b) Approaches to manage PPH (c) Challenges of preventing and managing PPH; (d) Ways of supporting midwives to overcome the challenges of preventing and managing PPH in rural health care settings (Table 2).

**Table 1:** Demographic characteristics of the study participants

Characteristics of the participants	N	%
Female	15	100%
Age		
30–39	2	13.3%
40–49	5	33.3%
50–59	8	53.3%
Level of Midwifery Education		
Certificate	12	80.0%
BSc	3	20.0%
Length of Midwifery practice		
3–4 years	2	13.3%
5–6 years	2	13.3%
Over 10 years	11	73.3%
Current area of practice		
Labour and Birth	2	13.3%
Antenatal Care, Labour, Birth & Postnatal care	13	86.7%

**Table 2:** Themes, categories and sub-categories from midwives’ experiences

	Themes	Categories	Sub-Categories
1	Interventions for preventing PPH	Antenatal care interventions Interventions for preventing PPH during labour and childbirth	Antenatal assessment, diagnosis and treatment of anaemia Antenatal education and health promotion
2	Approaches to PPH management	Medical care Emotional care	
3	Challenges associated with prevention and management of PPH	Issues with access to maternity care Effects of inadequate resources on prevention and management of PPH	
4	Ways of supporting midwives to overcome the challenges of preventing and managing PPH in rural health care settings		

### Theme 1a: Antenatal care interventions for preventing complications of PPH

Antenatal care provided opportunities for assessing and promoting maternal well-being in general, and preventing PPH through assessment, diagnosis and detecting risk factors associated with the development of anaemia during pregnancy. This category contains two sub-categories:

1. *Antenatal assessment, diagnosis and treatment of anaemia*

Women were encouraged to book early to enhance early investigations and identifications of risk factors for PPH such as anaemia. Full blood counts were routinely tested to assess and monitor haemoglobin as well as correcting anaemia in pregnancy to prevent complications of severe haemorrhage. In addition, folic acid supplementation was recommended throughout pregnancy to prevent folic acid deficiency. The combined use of folic acid and iron tablets helped to prevent iron deficiency during pregnancy. Women were also informed that folic acid can also help prevent congenital anomaly such as neural tube defect, which may develop in the first trimester.

“The rate of anaemia in Nigeria is alarming and killing many people. We do routine laboratory investigations of clients’ HB during pregnancy. Many women have low HB and low PCV because of poor diet. Women with low HB and low PCV levels are treated for anaemia with iron therapy during pregnancy”

“Antenatally, I encourage pregnant women to book on time at gestational age of 12-16 weeks. I check the level of the women’s PCV, that is, packed cell volume. We give them advise according to the results”.

The midwives also discussed the importance of prophylactic treatment of malaria.

“We give presumptive treatment to prevent malaria during pregnancy because malaria can lead to anaemia".

The midwives emphasized that since PPH is not always predictable, efforts should be made to maintain optimum haemoglobin levels during the antenatal period to enhance women’s abilities to cope with PPH if it occurs.

“It is not always easy to predict who will have PPH based on risk factors because few women who had PPH had no risk factor. It is better to make efforts during pregnancy to improve women’s HB level before labour so that her blood loss during childbirth will not have bad effects on their health”.

2. *Antenatal education and health promotion.*

Antenatal visits provided opportunities for health education aimed at promoting women’s health and wellbeing during pregnancy. The importance of eating healthy diet was discussed as well as the benefits of giving birth in health facilities.

“PPH is prevented by educating the mother from antenatal clinic during first, second and third trimesters on how to promote and maintain good health during pregnancy. Health education is also provided to the women on the importance of hospital birth”

"Antenatal health education is a very good tool. I advise women to eat a balanced diet to prevent anaemia."

Women were also given advice on preparation for labour and childbirth. The midwives provided women with information about active management of the third stage of labour to prevent PPH.

“To prevent PPH in my community setting we use active management of third stage of labour, especially when they have risk factors. We have proper health education during antenatal care. We prepare them for labour and birth during the antenatal clinic, health talk and training.”

Some midwives were unable to provide adequate antenatal health education to some women because of poor maternal health literacy, language barrier, personal, religious and spiritual beliefs held by the women. Health care staff with bilingual abilities and other voluntary translators acted as informal interpreters because none of the health facilities had professional translators.

“The hindrances we do have for effective prevention of PPH in the community clinic are some patients’ attitudes to accepting antenatal talk on healthy diet in pregnancy due to their religious beliefs and status which may result in poor feeding and anaemia. Some women are also reluctant to accept family planning talk antenatally. They don’t know the dangers of uterine atony due to multiparity”.

“Illiteracy of some women can be challenging. Some believe that PPH is caused by an enemy or witchcraft. It is difficult to explain to such people the actual cause of PPH when they have a very strong belief that it was caused spiritually by an enemy”

### Theme 1b: Intervention for preventing PPH during labour and childbirth

Midwives employed a variety of measures when managing all stages of labour to minimise the risks of PPH. They also identified the importance of managing physical labour pains as well as providing emotional support. Pharmacological and non-pharmacological pain management options were used to promote women’s comfort.

Efforts were made to minimize the risks of bleeding from perineal trauma by guarding the perineum during childbirth, minimising the use of episiotomy and timely suturing of the perineum. The clinical skills and experiences of the midwives contributed to effective management of the various stages of labour. In addition, qualified and experienced midwives were able to carry out manual removal of placenta using sterile gloves.

“How I manage 2^nd^ and 3^rd^ stages of labour in labour ward is important. Labour pain is not an easy one, so I always encourage the women not to bear down before time. I relief them from pain a bit with sacral massage, telling the woman stories, reassure her and give intramuscular analgesia. I always guard the perineum during delivery to prevent tear and bleeding”.

“I eliminate or at least minimise the use of episiotomy“. I suture any tear sustained or episiotomy given soon after the baby is born. This helps to reduce bleeding from any perineal tear that requires suturing. All stages of labour can be well managed with experience and these actions help to prevent PPH”.

The third stage of labour was actively managed by all participants using uterotonic agents such as ergometrine, syntocinon and misoprostol. The importance of accurate assessment of risk factors associated with PPH, accurate estimation of blood loss and careful monitoring of the woman’s condition and side effects of medication were discussed.

“In order to prevent this deadly condition (PPH), I try to accurately assess the risk factors and blood loss. Our health care facility guidelines encourage the practice of active management of third stage of labour. This has helped to reduce the number of PPH cases in our health care facility. I administer a uterotonic drug immediately after the birth of the baby. I also use controlled cord traction when delivering the placenta. We use oxytocin as our first choice of drug.

“What helps me to prevent PPH is the use of misoprostol, good history taking and careful conducting of delivery”

### Theme 2: Approaches to PPH management

The midwives’ understanding of PPH as an emergency, frightening and life-threatening condition enhanced their abilities to provide prompt medical and emotional care to the women and their birth partners to reduce the risks of complications and emotional distress. The midwives also discussed the challenges they experienced when managing PPH. Thus, the theme contains two categories: (a) Medical care following PPH and (b) Emotional support.

#### Medical care

The midwives identified PPH as an obstetric emergency that required prompt recognition, diagnosis and treatment to control the bleeding and prevent deterioration. The participants explained that having guidelines and policy for preventing and managing PPH enabled them to diagnose and manage PPH in a timely and effective manner.

“We used and followed the algorithm for management of PPH. This helped us to diagnose and treat PPH without wasting time. PPH was mainly caused by atonic uterus. Sometimes the steps in diagnosing and managing PPH happen at the same time. Having another midwife or doctor in the room to help was very useful”.

“PPH can be deadly, so we treat it as an emergency and try to implement the health facility protocol in a manner similar to cardiac arrest protocol, with the same attention to detail and documentation”

The midwives’ abilities to diagnose and treat PPH effectively were also influenced by their levels of knowledge, skills, clinical experiences and availability of resources including staff.

“I received adequate education and training on PPH management during my education program. I am a registered nurse and midwife. I have had a lot opportunities to work with more qualified midwives and learnt from them. The knowledge and experiences I gained have helped me to effectively manage PPH as a qualified nurse and registered midwife.”

#### Emotional care

The midwives spoke about PPH as a frightening condition requiring medical, emotional, and spiritual considerations and care. The importance of providing care in a competent, compassionate and sensitive manner to reduce anxiety and psychological distress was emphasized.

“PPH is an emergency, I try to be tactful and confident when the woman is bleeding heavily because the patient is always anxious and afraid of what might happen to her. I render care and reassurance in sensitive and skilful ways.”

The midwives discussed the importance of actively communicating with the woman and her partner to enhance their active participation in their care and alleviate their anxiety. The midwives demonstrated their abilities to pay attention to them and show empathy in the given situations.

“I always involve the woman in her care. I talk with her and draw her attention to what is happening before starting any intervention. I explain everything to her and make sure she understands what is happening and what we want to do to make her feel better”.

Religious and spiritual beliefs are sources of inner strength for many individuals. Severe PPH may disrupts these.

“PPH is an obstetric emergency and time is everything, but by God’s interventions and careful detection of the cause of the bleeding, we were able to control the bleeding”.

### Theme 3: Challenges associated with prevention and management of PPH

Prevention and management of PPH posed considerable challenges to the midwives in rural clinical setting and communities. Hindrances to the provision of effective care of PPH included access to maternity care and inadequate resources for managing PPH. Ways of overcoming these challenges in rural health care settings were also discussed.

Problems with access to maternity care centres resulted in delay in treatment of women. These include difficulties with transport and late referral from maternity home to health care facilities.

“Late referral from maternity home is not helpful. It leads to delay in treating the woman. The day we had PPH case in our facility, actually it was referred from a nearby maternity home”.

Inadequate resources were identified as a major barrier to the midwives’ efforts in adequate prevention and management of PPH. Insufficient resources such as infrastructure, labour ward equipment, laboratory facilities, medication, staff shortages were inhibitory factors for the provision of timely and adequate care. The government provided some medications and gloves, but medications were insufficient. Women and their relatives paid for the medications used to prevent and treat PPH.

“What inhibits my ability to effective prevention and management of PPH in health facility is that we don’t have adequate equipment and drugs. We don’t have lab for grouping and cross matching. We don’t have enough staff in the facility. Imagine in the health facility you see only one midwife doing everything alone, and you just have a maternity care attendant to help you”

“It was not easy managing PPH in the local area where there is no adequate equipment, drugs and staff. Many midwives and other health care workers don’t want to go to rural areas to work because there is no road, no electricity, no properly equipped health care facility”.

Although effective management of PPH was challenging, the midwives were motivated by the passion they had for their work, and determination to prevent maternal mortality and morbidity.

“Actually, it has not been easy managing PPH, but one feels satisfied when you have achieved your aim of controlling the bleeding and saving the life of the woman”.

### Theme 4: Ways of supporting midwives to overcome the challenges of preventing and managing PPH in rural health care settings

It is essential that midwives are adequately supported by their organisations and the government to overcome the challenges of inadequate resources including human resources.

“We will prefer to have a well-equipped labour room where we will have facilities that will enable us control bleeding for example, a bed where patients can be placed in a lithotomy position if necessary and placenta is expelled with its membranes completely with an angle light for easy assessment”

“Government should employ more trained midwives. We need more midwives in the labour ward. We also need enough equipment and drugs for managing PPH. More doctors should be assigned to work in health facilities in the rural areas”

Effective management of PPH in an on-going manner requires continuous practice review, emergency drills and continuing education and training of midwives. These actions are necessary for the provision of high standards of care to women everywhere.

“Just as with other advanced life support protocols, it is important to have a periodic review of everybody’s practice. We also need practice drills. Practice review is helpful in improving practice.

“I have gone to various training such as LSS training, that is, lifesaving skills; Mc pop training, that is, mandatory continuing professional development programme; Delivering Effective and Quality Health Service training in Nigeria (DEAQHSN); A Pivot to Adequate Health Care at the Grassroot (PAHCG). These training programs occur once every three to four years”.

Effective management of massive PPH is dependent upon a timely collaborative multidisciplinary team approach. Midwives are therefore encouraged to escalate care in a timely manner to their senior colleagues and other multidisciplinary team members.

“As a midwife, I was involved with the multidisciplinary team including obstetricians and anaesthetists in looking after patients diagnosed to have intractable post-partum haemorrhage and not managed with standard medical treatment and were subsequently treated with operative interventions. The operative interventions were successful”

“Midwives should not delay transferring care to doctors if they can’t control the bleeding. More studies are required on how to recognise and rectify any deficiencies in midwifery practice for urgent transfer of patients for surgery if indicated. If bleeding cannot be managed by midwives, they must transfer to doctors because surgery may be required to save the woman’s life”.

## Discussion

The quality of care during pregnancy, labour and childbirth contributes to maternal health and wellbeing. Prevention of anaemia during pregnancy is crucial in minimizing potential morbidity and mortality associated with PPH [9, 13, 14]. Reluctance to accept antenatal education was attributed to poor maternal health literacy, language barrier, personal and spiritual beliefs of some women, factors responsible for maternal anaemia in Nigeria [33].

Nigeria is a multicultural, multireligious and multilingual country. To be able to provide holistic women-centred maternity care to all women, it is important that midwives understand and appreciate the roles of culture, religion, and spiritual beliefs of many individuals in Nigeria [34, 35]. Being culturally competent will enable midwives to understand, appreciate and interact with women from different cultural and religious backgrounds [35, 36]. Using professional interpreters during antenatal education will also help alleviate language and communication difficulties, experienced by both midwives and women [37].

Studies have found that (prophylactic) treatment for malaria in countries with moderate to high prevalence of malaria helps to inhibit or minimize the effects of malarial parasites on the red blood cells, thus reducing the risks of maternal anaemia, placental malaria, preterm birth and pregnancy loss [1, 38, 39]. To minimize the risks of poor compliance, women should be given options of malaria treatment with minimal side effects [38].

Clinical skills and continuity of midwifery care enabled midwives to use strategies such as guarding the perineum during childbirth, minimising the usage of episiotomy and timely suturing of the perineum to reduce bleeding [40, 41]. Lack of access to oxytocin for active management of third stage resulted in the use of misoprostol. Although misoprostol is not as effective as oxytocin, it is mostly used as first line drug of choice in low socioeconomic settings because it is less expensive and can be readily stored [42]. Misoprostol given by trained health care workers is effective and safe for the prevention of PPH [43]. Although oxytocin has great impact in reducing PPH, its quality and impact on PPH decreases because of its sensitivity to heat and the need of appropriate storage in a cold chain [44]. Uterotonic agents in Nigeria where there is generally hot weather together with unstable electricity have implications for effective management of PPH especially in rural communities. The WOMAN Tranexamic trial has shown that tranexamic acid (TXA) helps in reducing bleeding-related maternal deaths [45]. As an antifibrinolytic pharmacological agent, TXA is best used with oxytocin for prevention of PPH [46].

Studies have shown that it is important to utilise timely holistic, multidisciplinary team approaches to prevent physical deterioration and psychological distress when managing life-threatening complication in childbirth [19, 20, 47]. It has been suggested that having positive mental attitudes despite insufficient resources enhances growth of passion and work engagements, job satisfaction and well-being, promoting midwives’ effectiveness at work [48].

Timely maternal care provision in Nigeria is hindered by delay of women in making decisions to seek maternity care, delay in locating or arriving in health facilities, and delay in receiving care from skilled health care professionals on arrival [20, 22, 23]. It is therefore important to improve access to maternity care, trained skilled health care professionals in the 40 primary health facilities and secondary health facilities to enhance equitable access to good quality care for these women [20]. At present, the authors are uncertain of the exact number of all the health care professionals in the LGA because of constant migration of staff from the rural communities to the cities. This is a limitation of our study.

Although the midwives acknowledged having adequate knowledge and skills required to manage PPH, they also identified the need for continuing professional education and training, emergency drills, and continuous practice review to enhance their abilities to provide high standards of maternity care. This could be achieved by promoting lifelong learning and midwives’ continuing professional development using a variety of learning strategies involving theoretical inputs, clinical and reflective practice, and quality improvement initiatives [49].

## Conclusion

Post-partum haemorrhage is a leading cause of maternal death in Nigeria. Exploring maternity care experiences with midwives is important to identify ways of improving maternal health and reducing death associated with PPH. Antenatal education, assessment of maternal health and wellbeing, diagnosis and treatment of anaemia were strategies during the antenatal period. Holistic care and timely multidisciplinary team approaches facilitate effective management of PPH. Barriers include staff shortages, insufficient equipment and medications, and delay of arriving in maternity care centres.

Poor maternal health literacy, language barriers, personal and spiritual beliefs may hinder effective antenatal education, needing culturally competent midwives when providing care to women from different cultural and religious backgrounds. Suitable professional interpreters need to be provided to enhance communication. There is a need for the government to provide adequate resources for preventing and managing PPH in rural health care settings to reduce the risks of maternal morbidity and mortality.

## Supplementary Information

**Additional file 1.** Interview guide for post-partum haemorrhageinterviews.

## Data Availability

The dataset used and analysed during the current study are available from the corresponding author, f.kalu@qub.ac.uk on reasonable request.

## Abbreviations

PPH: Postpartum haemorrhage
WHO: World Health Organisation
LMIC: Low and Middle-Income Countries
MMR: Maternal Mortality Ratio
LGA: Local Government Area
