# Supplementary material for: Midwives’ experiences of reducing maternal morbidity and mortality from postpartum haemorrhage (PPH) in Eastern Nigeria
Source: BMC Pregnancy Childbirth. 2022 Jun 8;22:474. doi: 10.1186/s12884-022-04804-x (PMC9175340; doi:10.1186/s12884-022-04804-x)
Supplement: Supplementary file 1 — Additional file 1. Interview guide for post-partum haemorrhageinterviews. [file 12884_2022_4804_MOESM1_ESM.doc]

**Interview guide for post-partum haemorrhage interviews**

Warm up

We appreciate you giving up time to attend this interview. We (FAK) and (JNC) are midwife lecturers and researchers. We are interested in healthcare in general, and maternity care in particular. We are interested in exploring your experiences in preventing and managing PPH in your clinical setting/community. There is no right or wrong answer. Confidentiality will be maintained. Is there any question you would like to ask? Just let me (FAK) know.

Question one

Could I start by asking you to tell me about your experiences of providing care to women who had post partum haemorrhage.

What was it like to look after these women?

How did you manage third stage of labour?

What guideline/protocol guides your practice in relation to PPH prevention and management?

How do you prevent post partum haemorrhage in your community/clinical setting?

Question two

How were you prepared to be able to look after these women?

What learning opportunities were available to prepare you for the provision of care to these groups of women?

How adequate was your preparation?

Was there any particular gap or area where further preparation was required?

Question three

What support system was in place for you within your organisation/clinical area that helped you to provide care to these women?

What type of additional support would you like to have from your organisation/community to meet this crucial aspect of your professional role?

Question four

What other things help you to effectively prevent and manage PPH in your clinical setting/community?

What other things inhibit your ability to effectively prevent and manage PPH in your clinical setting/community?

Question five

Have you any other suggestion on how PPH can be effectively prevented and managed in your clinical setting/community?

Thank you for your time and interest in this research.
